# Atlanta Medical College—The War

**Published:** 1861-06

**Authors:** 


### Atlanta Medical College—The War.

From the opening of the present Course of Lectures, on first Monday in May, to within a few days ago, the Faculty and Class entertained the hope of having, irregularly, the services of Dr. W. F. Westmoreland, Prof. of Surgery. That hope has fled. The removal of the 3rd Georgia Regiment of Volunteers, to which he is attached as Surgeon, from Pensacola to Virginia, makes it impossible for him to be with us any more during the session. The three days spent at home, while the Regiment was in transit to the new place of destination, commenced and closed his connection with the present class. While we, in common with other members of the Faculty and the Class, feel sensibly the loss we sustain in his absence—and more, the fraternal ties which connect us with an only brother—we have no complaint to make—no objection to urge. The cause in which he has embarked is the cause of our country—the cause of justice and humanity. With pride we see and feel the cheerfulness with which this sacrifice is made to the cause of our beloved land. While the absence thus caused entails double labor on another member of the Faculty, that labor is being willingly undergone by Dr. Brown, the Prof. of Anatomy, and, we think, to the entire satisfaction of the Class. Indeed the spirit of '76—that spirit which in our revolutionary fathers, resisted injustice and oppression—pervades the Institution; so much so, that while with difficulty other members of the Faculty can restrain their inclinations sufficiently to remain during the session, the Class of Students have organized for military drill, occasionally, to answer the place of physical exercise, as well as to qualify them for the emergencies of war, should circumstances make it necessary to go into military service when they return home.

The Class, though smaller than usual, owing to the large number of Medical Students volunteering, is nevertheless respectable in number, and high in moral and intellectual qualities. Many we know, who intended coming, have volunteered, and are now zealously engaged in the defense of our rights on the tented field. When we see young men who have commenced the study of a profession, and, in the midst of their favorite pursuit, lay down the scalpel and seize the sword, we think such men cannot be successfully resisted—such a people cannot be conquered.

